# Environmental F actors coordinate circadian clock function and rhythm to regulate plant development

**DOI:** 10.1080/15592324.2023.2231202

**Published:** 2023-07-23

**Authors:** Ying Zhang, Yuru Ma, Hao Zhang, Jiahui Xu, Xiaokuan Gao, Tengteng Zhang, Xigang Liu, Lin Guo, Dan Zhao

**Affiliations:** a College of Life Sciences, Hengshui University, Hengshui, Hebei, China; b Institute of Biotechnology and Food Science, Hebei Academy of Agricultural and Forestry Sciences, Shijiazhuang, China; c College of Life Sciences, Hebei Normal University, Shijiazhuang, Hebei, China

**Keywords:** Circadian clock, environmental factors, growth and development, agricultural production

## Abstract

Changes in the external environment necessitate plant growth plasticity, with environmental signals such as light, temperature, and humidity regulating growth and development. The plant circadian clock is a biological time keeper that can be “reset” to adjust internal time to changes in the external environment. Exploring the regulatory mechanisms behind plant acclimation to environmental factors is important for understanding how plant growth and development are shaped and for boosting agricultural production. In this review, we summarize recent insights into the coordinated regulation of plant growth and development by environmental signals and the circadian clock, further discussing the potential of this knowledge.

## Introduction

### The concept of the circadian clock

To modulate their growth and development in response to changes in the natural environment, plants must accurately perceive environmental cues such as light and temperature. The circadian clock in plants is an endogenous, cell-autonomous regulatory system that generates a nearly 24-hour oscillation influenced by environmental signals. Its conversion of environmental signals into temporal information leads to many changes, such as alterations in transcriptional and post-transcriptional rhythms to synchronize metabolic, physiological, and biochemical programs with the external environment.^1^ Therefore, the circadian clock integrates intrinsic environmental signals (such as light and temperature) to coordinate plant metabolism and development for acclimation to the surroundings.^2,3^

The plant circadian clock comprises three major components: the input pathways transmitting perceived environmental signals; the oscillator composed of various transcription-translation feedback loops; and output pathways that couple the environmental signals with plant biology, resulting in a coordinated rhythm between cellular behavior and environmental signals.^4^ As the expression of core clock genes is affected by upstream input pathways, we will first introduce the factors comprising the circadian oscillator, before discussing the upstream input pathways and downstream output pathways.

## The core oscillator

**Figure 1.**
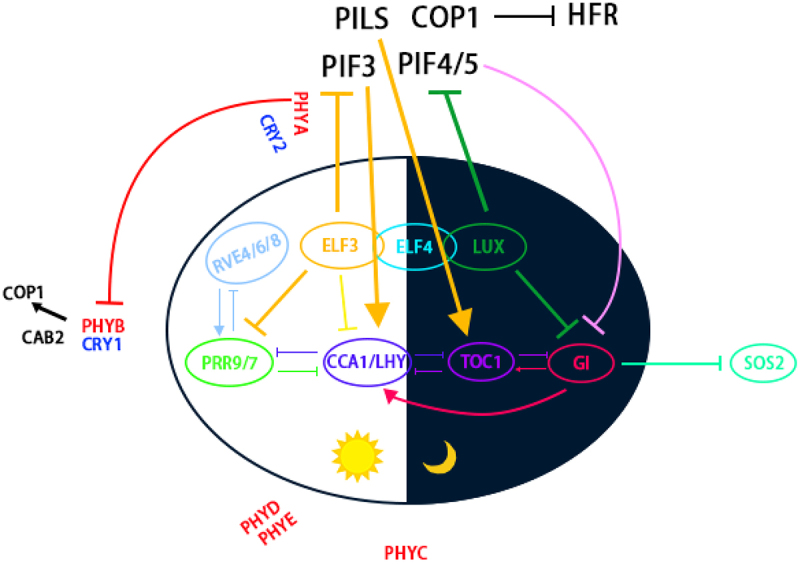
Environmental factors coordinate the circadian clock to regulate plant development.

The circadian system is a complex signaling network controlled by at least 20 genes in Arabidopsis (*Arabidopsis thaliana*). At the center of the circadian system is a core oscillator responsible for generating circadian rhythms.^5,6^ Three closely related transcription-translation feedback loops make up the core oscillator: morning, central, and evening loops, associated with the key transcription factors LATE ELONGATED HYPOCOTYL (LHY), CIRCADIAN CLOCK ASSOCIATED 1 (CCA1), and TIMING OF CAB2 EXPRESSION 1 (TOC1, also known as PSEUDO-RESPONSE REGULATOR 1 [PRR1]), respectively (Figure 1).

Genes encoding the core oscillator can be divided into early cycle, central cycle, and late cycle genes according to their peak expression window. *CCA1* and *LHY* show their highest expression at dawn and their encoded proteins form heterodimers (CCA1–LHY) *in vivo*, which directly bind to the evening element in the *TOC1* promoter to repress *TOC1* transcription. *TOC1* was the first evening-expressed gene whose transcription was shown to be inhibited by CCA1–LHY. Furthermore, TOC1 directly binds to the *CCA1* promoter and inhibits *CCA1* expression, and CCA1 and LHY can inhibit each other’s expression.^7^ Single mutants of *CCA1* or *LHY* have shorter circadian periods, while the *cca1 lhy* double mutant has very short period with low amplitude, thus presenting a highly disordered rhythm. CCA1 and LHY also inhibit the expression of other evening genes such as *GIGANTEA* (*GI*), *LUX ARRHYTHMO* (*LUX*), *EARLY FLOWERING 3* (*ELF3*), and *ELF4*.^8^

Mutual inhibition among CCA1, LHY, and TOC1 requires pseudo-response regulators (PRRs) to maintain proper rhythmic timing between morning and evening genes. While CCA1 and LHY inhibit *PRR5* transcription,^9^ they also activate *PRR9* and *PRR7* transcription. Peak accumulation of PRR9, PRR7, PRR5, PRR3, and PRR1 occurs in succession within a 12-hour period from dawn to dusk, with their accumulation inhibiting *CCA1* and *LHY* transcription. Following peak expression of *CCA1* and *LHY* in the morning, *PRR9* expression peaks, followed by expression of the afternoon-expressed genes *PRR7*, *PRR5*, and *PRR3*, and finally the evening gene *TOC1*.^10^ This perpetual inhibition mechanism maintains low expression of *CCA1* and *LHY* before dawn. PRRs also inhibit expression of *REVEILLE8* (*RVE8*), which encodes a MYB-like transcription factor similar to CCA1 and LHY, involved in the circadian clock.^11^ In addition, the TCP (Teosinte branched 1, Cincinnata, PCF) transcription factor CCA1 HIKING EXPEDITION (CHE) inhibits *CCA1* transcription by interacting with TOC1.^12^ CCA1 and LHY activate the expression of *PRR7* and *PRR9* genes, whereas PRR7 and PRR9 inhibit *CCA1* expression, forming a morning feedback loop (Figure 1).

LUX, ELF3, and ELF4 form the evening complex (EC), which directly suppresses the morning gene *PRR9*. Expression of *GI* is suppressed by CCA1 and LHY in the early morning;^13^ however, GI is required alongside TOC1 for maintaining the circadian rhythm at night.^14^ The EC and TOC1 are also involved in CCA1- and LHY-mediated inhibition of *GI*.^15^

## The input pathways

The input pathways in the circadian system transmit perceived environmental signals to the core oscillator and regulate the expression of circadian clock genes, setting the speed and phase of the clock. In contrast to its behavior under diurnal conditions, the circadian oscillator does not always exhibit an exact 24-hour period when released into constant conditions (those lacking entraining cues such as light-dark and/or temperature cycles). To produce an accurate 24-hour period, the internal oscillator must be synchronized with the external environment. The main environmental signals used to synchronize the circadian clock are light and temperature.^2^ Signaling cascades initiated from cryptochrome and phytochrome family members mediate the induction of *CCA1*, *LHY*, *PRR9*, and *GI* transcription. After light activation, photoreceptors migrate to the nucleus and interact with PHYTOCHROME INTERACTING FACTOR 3 (PIF3) to form a ternary complex; this induces the expression of *CCA1* and *LHY* by binding to G-box elements in their promoters. Calcium (Ca^2+^) and calmodulin signals are also involved in the response of the biological clock to light,^16^ while light controls the degradation of PRR5, PRR7, PRR9, TOC1, and GI.^17–19^ In turn, some clock genes are also involved in light-mediated pathways. For example, GI plays a role in phytochrome B (phyB)-mediated development and permits light to influence the circadian clock.^20^

## The output pathways

The output pathways couple environmental signals with biological programs to control plant growth and development, photosynthesis, flowering time, biotic and abiotic stress responses, and plant hormone metabolism, to name a few. The mechanisms by which the circadian clock regulates flowering time and hypocotyl elongation are well known.^21^ In Arabidopsis, the transcription factor CONSTANS (CO) plays a central role in sensing day length to regulate flowering. *CO* is transcribed in the evening, and CO protein is rapidly degraded by the E3 ubiquitin ligase CONSTITUTIVELY PHOTOMORPHOGENIC 1 (COP1) in the dark, which inhibits flowering under short days.^22^ *COP1* expression peaks in the daytime; however, under long-day photoperiods, COP1 activity is inhibited by cryptochromes, allowing accumulation of CO. This induces expression of *FLOWERING LOCUS T* (*FT*) and *TWIN SISTER OF FT* (*TSF*) to promote flowering.^23,24^ Thus, photoperiodic regulation of flowering is mediated by endogenous rhythmic proteins (CO) and external signals (light).

PIF4 and PIF5 promote hypocotyl growth. The EC inhibits *PIF4* and *PIF5* expression at night and promotes *PIF4* and *PIF5* expression in the early morning.^13,25^ In addition, phyB promotes PIF4 and PIF5 degradation under red light to prevent hypocotyl elongation.^26^ The circadian system therefore affects PIF4 and PIF5 accumulation to regulate hypocotyl elongation.

## Crosstalk between the circadian clock and light

Light is critical for the regulation and maintenance of circadian rhythms in plants. Changes in photoperiod affect the phase of the circadian clock and alter plant growth, development, and metabolism. Photoreceptors, such as cryptochromes, phytochromes, and ZEITLUPE (ZTL) family members, perceive light signals and transmit this information to the central circadian oscillator through various mechanisms.^27^ The signals generated by different photoreceptors can interact and integrate.^28^

Photoreceptors are involved in the dose-dependent perception of far-red, red, blue, and ultraviolet lights^29^ and mediate seed germination, photomorphogenesis, photoperiodism, temperature responses, phytohormone metabolism, and epigenetic modifications. Cryptochrome, the first blue light receptor to evolve in plants, is ubiquitous in plants^30^ and participates in regulation of the circadian clock, photomorphogenesis, stomatal opening and closing, flowering, and other growth and developmental programs.

## Phytochrome cooperates with the circadian clock to regulate plant development

Under red light, the pace of the circadian clock is preserved by the red/far-red photoreceptors phytochrome A and B. Compared with wild-type plants, the circadian period of the Arabidopsis *phyA* mutant is longer under weak red light (<1 μmol/m^2^/s), while that of the *phyB* mutant is longer under moderate red light (>5 μmol/m^2^/s). The circadian period of these mutants is shortened by high-fluence red light.^31^ The circadian period of the *phyABCDE* quintuple mutant becomes only slightly shorter with increasing light intensity; however, plants overexpressing *PHYB* display a light-dependent acceleration of circadian pace^32,33^ indicating that phytochromes play an important role in maintaining circadian period under red light. The *phyB* mutant also has a shorter circadian period under white light conditions.^34^ These findings indicate that strong red light shortens the circadian period, while weak light (or far-red light) lengthens the circadian period.

phyB interacts directly with key circadian clock factors including CCA1, LHY, GI, TOC1, LUX, and ELF3, with the interaction between phyB and CCA1, TOC1, and LUX being regulated by red/far-red light. This observation may partly explain the relationship between light signals and the circadian clock.

Light signals are also integrated with the circadian clock through cryptochromes. The circadian period of Arabidopsis *cry1* and *cry2* mutants is longer under continuous blue light.^31^ The longer circadian period of the *cry1* single mutant is not dependent on blue light intensity, while weak blue light induces a slightly shorter circadian period in *cry2* single mutants than in wild-type seedlings. However, the *cry1 cry2* double mutant has a longer circadian period under all fluences of blue light, suggesting that CRY1 and CRY2 have redundant roles in Arabidopsis.^29^ Compared with *phy* and *cry* mutants, *ztl* mutants have a longer circadian period under both continuous light and constant darkness, and overexpressing *ZTL* causes an arrhythmic phenotype, indicating that ZTL not only mediates input of external light signals, but is also a key factor in the core oscillator.^35^

Although the role of phytochromes and cryptochromes in coordinating external light signals with the internal circadian clock is well known, it is not clear how the light signals transduced from these photoreceptors affect the circadian clock. COP1 interacts with ELF3 to degrade GI through the 26S proteasome pathway.^36^ The E3 ubiquitin ligase activity of COP1 is also inhibited by phytochromes and cryptochromes in a light-dependent manner.^37^ Since ELF3 and GI are involved in circadian clock regulation, COP1 may function as a “direct connection” between the light signal and the circadian clock. Light-activated phyB maintains a strong circadian rhythm in the nucleus even in the absence of photosynthesis and other light-activated photoreceptors.^38^ Moreover, phyB inhibits the activities of COP1 and PIFs in the nucleus. Although *PIF3* overexpression or deletion does not affect the circadian clock, most PIFs interact with TOC1,^39,40^ suggesting that PIFs have an important role in integrating external light signals with the circadian clock. Furthermore, the expression of many key circadian clock genes is induced by light.^41^ Among them, *ELF4* is regulated by three positive regulatory factors, ELONGATED HYPOCOTYL 5 (HY5), FAR-RED ELONGATED HYPOCOTYL 3 (FHY3), and FAR-RED IMPAIRED RESPONSE 1 (FAR1), in the phyA signaling pathway, which directly bind to the *ELF4* promoter and induce its expression during the day.^42^ Because the protein level of HY5 is regulated by COP1, it is possible that phyA as well as other phytochromes or cryptochromes induce *ELF4* expression in the light by stabilizing HY5.

In contrast with phytochromes and cryptochromes, ZTL family members directly mediate the expression of key circadian clock genes. ZTL directly interacts with PRR5 and TOC1 in a light-dependent manner to promote their degradation,^43^ while the interaction between PRR3 and TOC1 inhibits the ZTL-mediated degradation of TOC1 in the cytoplasm.^44^ Furthermore, PRR5 also interacts with TOC1 and promotes its nuclear translocation, thus inhibiting its ZTL-mediated degradation in the cytoplasm.^45^ These results show that ZTL-mediated light signal input mainly affects the circadian clock by regulating the protein level of TOC1.

## Cryptochromes cooperate with the circadian clock to regulate plant development

CRY1 and CRY2 are blue and ultraviolet light receptors that have important roles in regulating the circadian clock oscillator in Arabidopsis. The mechanism of blue light signal transmission to the core oscillator is not well understood. CRY2 interacts with PIF4 and PIF5 to bind to chromosomes in Arabidopsis.^46^ PIF4 concatenates the circadian clock with the red-light signal transduction pathway.^47^ PIFs interact with PRR9, PRR7, and PRR5, which inhibit the transcriptional activation activity of PIFs on pre-dawn genes.^48^ The late transcriptional complex, a key component of the circadian clock, binds to *PIF4* and *PIF5* promoters and regulates their expression, demonstrating the dynamic control of the circadian clock over hypocotyl growth.^25^ Although the roles of PIF4 and PIF5 in the circadian clock are well understood, a function for PIFs in blue-light-mediated regulation of the circadian clock input pathway has not been reported. Photoregulatory protein kinases (PPKs) regulate the period of the circadian clock under continuous red light.^49^ PPKs also regulate CRY2 activity under continuous blue light.^50^ However, it has not been reported whether PPKs regulate the circadian clock by modulating CRY2 activity. Weak Arabidopsis *cop1* mutants have a shorter circadian period,^51^ indicating that COP1 is also associated with circadian clock input pathways. COP1 and ELF3 regulate the stability of GI to mediate resetting of the circadian clock and photoperiodic flowering.^52^ Nevertheless, there are few reports on whether CRY2 mediates this input pathway.

## PIFs facilitate circadian clock regulation

PIFs are phytochrome-binding proteins containing a basic helix-loop-helix (bHLH) domain that function as transcription factors. They have key roles in light signal transduction from phytochromes^53^ and are also involved in pathways for transduction of many other signals, such as temperature signals, circadian clock signals, phytohormone signals, and biotic and abiotic stress signals. PIF1, PIF3, PIF4, and PIF5 contain common and unique DNA binding sites. Because of their different expression patterns, *PIF1*, *PIF3*, *PIF4*, and *PIF5* are presumed to perform similar but different biological functions. *PIF1*, *PIF4*, and *PIF5* expression is regulated by the circadian clock, and PIF4 regulates hypocotyl growth via the circadian clock. Although *PIF3* expression is irregular, PIF3 protein accumulation shows clear diurnal rhythmicity, being high at night and low during the day. Phytochromes are negatively involved in light signal transduction; therefore, *phy* mutants show a longer circadian period owing to diminished red light input. If PIFs are also involved in light signal transduction to the circadian clock via phytochromes, then *pif* mutants should show a short circadian period phenotype. However, the *pifQ* (*pif1 pif3 pif4 pif5* quadruple) mutant exhibits a longer circadian period,^54^ suggesting that PIFs are not directly involved in light signal input to the circadian clock. PIFs do play crucial roles in the input pathways linking metabolic signals to the circadian clock,^54^ and binding of PIFs to the *CCA1* and *LHY* promoters is significantly increased by the addition of sucrose.

PIFs bind to the promoters of key circadian clock genes. For instance, PIF3 interacts with the circadian clock protein TOC1, whose encoding gene is expressed during the night; together, PIF3 and TOC1 bind to the promoters of genes expressed in the morning to regulate their expression.^39^ TOC1 inhibits the transcriptional activation activity of PIF3. This protein interaction results in transcriptional inhibition that inhibits plant growth when TOC1 accumulates to high levels at dusk; the inhibition is relieved near the early hours of the morning to promote growth. TOC1 also interacts directly with PIF4 to inhibit its transcriptional activation activity.^55^ These studies demonstrate that PIFs interact with the core components of the circadian clock, thus participating in circadian clock-regulated growth and development.

## The role of PIF4 in responding to circadian clock signals

The abundance of PIF4 is affected by a variety of environmental conditions and endogenous factors. In addition to being activated by high temperature, PIF4 is inhibited by phytochrome-mediated degradation and cryptochrome-mediated inactivation.^46^ Furthermore, phytochrome indirectly affects *PIF4* expression^56^ by mediating the formation of the EC consisting of ELF3, ELF4, and LUX, which binds to the *PIF4* and *PIF5* promoters to inhibit their expression. In the morning, as the level of the EC steadily decreases, expression of *PIF4* and *PIF5* steadily increases, peaking at noon, when the level of the EC is lowest.^25^ PIF4 and PIF5 are unstable in the light, suggesting that the amount of endogenous PIF4 and PIF5 is highest in the evening, when *PIF4* and *PIF5* expression is still relatively high and their proteins are stable.^57^ However, one study found that, contrary to expectations, PIF4 is maintained at a high level during the day under short-day conditions.^58^ Similar to the mechanism of PIF3, the DNA binding ability of PIF4 is thought to be inhibited by active phyB in the light.^46,59^ Moreover, low levels of gibberellic acid in the light further inhibit PIF4 DNA binding ability as a result of DELLA protein accumulation.^60^ The interaction between CRY1 and PIF4 induced by blue light also inhibits the transcriptional activation activity of PIF4.^61^ Therefore, phyB, DELLA, and CRY1 jointly ensure that PIF4 is maintained at a low level during the day to inhibit hypocotyl elongation.

## Crosstalk between the circadian clock and temperature

Similar to light, temperature is a major external environmental signal that mediates plant growth and development and also serves as an input signal to regulate the internal circadian clock. Temperature signals alter the circadian clock cycle to adjust plant growth and development. The circadian periods of plants with mutations in key circadian clock genes, such as *PRR7*, *PRR9*, *GI*, and *RVE8*, remain relatively constant across a range of physiologically relevant temperatures, which is known as temperature compensation.^62^ However, the *prr7 prr9* double mutant shows a temperature compensation defect, with extreme lengthening of circadian period at higher temperature. Silencing of *CCA1* and *LHY* in the *prr7 prr9* double mutant background completely restores temperature compensation, indicating that *PRR7* and *PRR9* regulate *CCA1* and *LHY* activity in response to temperature changes.^63^ Temperature signals coordinate the circadian clock through transcriptional and post-transcriptional regulatory mechanisms. For example, *C-REPEAT/DRE BINDING FACTOR 1* (*CBF1*) is induced by low temperature (4°C), and CBF1 binds directly to the *LUX* promoter to regulate *LUX* expression.^64^ FLOWERING BHLH 1 (FBH1), a bHLH transcription factor, binds directly to the *CCA1* promoter and regulates its rhythmic expression under high temperature.^65^ Temperatures within the normal range for plant growth (16–28°C) also affect the circadian clock. Many studies suggest that the temperature signal is integrated into the plant circadian clock through the EC. In *lux* mutants, GI shows no rhythm in response to hot/cold cycles, and the circadian clock in dark-grown *elf3* mutants does not respond to temperature signals.^66^ Furthermore, *PRR7*, *GI*, and *LUX* expression is induced by high temperature, but this induction is eliminated in *elf3*, *elf4*, and *lux* mutants,^15^ indicating that the EC plays a vital role in mediating external temperature signals and coordinating internal circadian clock signals. The role of phyB in perceiving temperature^67^ suggests that phyB integrates extrinsic light and temperature signals to affect the circadian clock. In summary, phyB and the EC integrate temperature and light signals to alter the circadian clock through a series of complex regulatory networks, allowing plants to adjust their growth, development, and metabolism to suit the external environment.

## Crosstalk between the circadian clock and auxin

Genome-wide transcriptome analysis showed that 90% of Arabidopsis genes exhibit some type of rhythm.^68^ Of these, at least 16% of all Arabidopsis genes are upregulated by the circadian clock. The circadian clock regulates the plant response to endogenous auxin, as well as the expression of multiple auxin signaling genes,^69^ indicating a crosstalk between the circadian clock and auxin signaling. Accumulation of the MYB-like transcription factor RVE1 is regulated by the circadian clock; RVE1 levels peak at dawn, decrease during the day, and increase at night. RVE1 positively regulates the auxin biosynthesis gene *YUCCA8(YUC8)* to increase the concentration of auxin at dawn.^70^

Auxin promotes *TOC1* expression in the basal meristem of lateral roots to promote lateral root growth. Deletion of *TOC1* inhibits lateral root development.^71^ The clock-related component TIME FOR COFFEE (TIC) regulates root meristem size. The loss-of-function *tic1* mutant shows decreased division of meristem cells and fewer and shorter root meristems. In the T-DNA insertion allele *tic2*, auxin levels in the root are lower relative to the wild type, resulting in decreased polar auxin transport and fewer root meristems.^72^ Furthermore, CCA1 binds to the *PICKLE* (*PKL*) promoter to promote its transcription. PKL regulates trimethylation of histone H3 at lysine 27 (H3K27me3) of the auxin-responsive genes *INDOLE-3-ACETIC ACID INDUCIBLE 19* (*IAA19*) and *IAA29* to activate their transcription and promote hypocotyl elongation.^73^ The expression of *PIF4* and *PIF5*, encoding transcription factors involved in hypocotyl elongation, is inhibited by the EC in the early evening.^74^ The interaction of PIF4 with the EC component ELF3 inhibits PIF4 activation of its target genes *PHYTOCHROME INTERACTING FACTOR 3-LIKE 1* (*PIL1*) and *XYLOGLUCAN ENDOTRANSGLYCOSYLASE 7* (*XTR7*) and promotes hypocotyl elongation at dawn.^75^

## The circadian clock regulates flowering time

The circadian clock regulates flowering time in Arabidopsis and many crops. In Arabidopsis, *cca1* and *lhy* mutants flower earlier under short-day (SD) conditions, while the *cca1 lhy* double mutant flowers earlier under both long-day (LD) and SD conditions. Overexpressing *CCA1* or *LHY* results in delayed flowering under LD and SD conditions in Arabidopsis.^76^ The *toc1* mutant also flowers earlier under SD conditions.^77^ The *prr5* single, *prr7* single, and *prr5 prr7 prr9* triple mutants exhibit delayed flowering under LDs, while *PRR5*- and *PRR9*-overexpression lines flower earlier under LD and SD conditions in Arabidopsis.^78^ *lux* mutants flower much earlier under SD than under LD conditions.^79^ ELF3 is also required for the photoperiodic response and flowering time in plants. ELF3 regulates flowering in Arabidopsis through the CO–*FT* pathway and the non-CO pathway.^80^ Indeed, *elf3* mutants flower much earlier under SDs than under LDs, essentially showing no photoperiod sensitivity (Zhao et al. 2020). *ELF3* or *ELF4* overexpression delays flowering under LD conditions^81^Zhao et al. 2020). Similarly, *elf4* mutants flower earlier, and *CO* expression increases significantly in these mutants under SDs, with no significant change in flowering time under LDs.^82^ A polymorphism in *PsHR* (*HIGH RESPONSE TO PHOTOPERIOD*), an ortholog of Arabidopsis *ELF3*, is associated with flowering time in pea (*Pisum sativum*). Pea varieties with nonfunctional *Pshr* alleles flower earlier under SD conditions.^83^ In soybean (*Glycine max*), the flowering time of *lhy1a lhy1b lhy2a lhy2b* quadruple mutants is delayed under LDs.^84^ GmPRR37 inhibits *GmFT2a* and *GmFT5a* expression to promote flowering, but promotes *GmFT1a* expression to inhibit flowering. Overexpressing *GmPRR37* delays flowering under LDs, while the *Gmprr37* mutant flowers earlier under natural LDs.^30^ The flowering of soybean *lux1 lux2* double mutants is more delayed under SDs than under LDs.^85^ Natural variation in the *GmJ* gene leads to altered flowering time. The flowering time of the near isogenic line NIL-*j* under SDs is later than that of NIL*-j*.^86^ In rice (*Oryza sativa*), OsPRR37 suppresses *Heading date 3a* (*OsHd3a*) expression to inhibit flowering under LDs. *Japonica* rice varieties harboring nonfunctional alleles for both *OsPRR37* and *Grain number, plant height, and heading date 7* (*Ghd7*) flower extremely early.^87^ Rice *Oself3* mutants exhibit a delayed heading date phenotype under LDs, but no significant change in heading date under SDs. Overexpressing *OsELF3* leads to early heading under LD conditions.^88^ In barley (*Hordeum vulgare*), flowering of varieties containing nonfunctional alleles of the *PRR7* ortholog *Hvppd-H1* (*Photoperiod-H1*) is delayed.^89^ The *Hveam10* (*early maturity 10*) and *Hveam8* mutants flower earlier under both LD and SD conditions.^90,91^ In wheat (*Triticum aestivum*), mutations in *Ppd-1a* on the A, B, or D genomes lead to different degrees of early flowering.^92^ In sorghum (*Sorghum bicolor*), SbPRR37 promotes the expression of the flowering suppressor gene *SbCO* and inhibits *SbFT* expression, thus inhibiting flowering under LD conditions. *SbPRR37* expression is suppressed in the dark, and therefore sorghum flowers under SD conditions.^93^

Researchers have long believed that the biological clock function of plants is uncoupled. Only a few studies have shown weak local coupling between cells. A comprehensive tissue-specific analysis of leaf tissue was conducted by implementing two new technologies, indicating that the vascular system and mesophyll clock of Arabidopsis are asymmetrically regulated. The circadian clock in the vascular system has different characteristics from other tissues. It circulates steadily without environmental clues and affects the regulation of the circadian clock in other tissues. In addition, it was found that genes rich in vascular systems with rhythmic expression were preferentially expressed in even numbered cells.^94^

Nitrogen (N) is an important nutrient that affects various developmental processes in plants. As flowering requires resources to develop sink tissue for reproduction, the availability of nutrients is closely related to this process. Low nitrogen levels accelerate the transition of flowers; The flowering BHLH4 (FBH4) transcription factor is a key regulatory factor for nitrogen responsive flowering in Arabidopsis. The early flowering induced by low nitrogen is damaged in the *fbh* quadruple mutant.^95^ High nitrogen fertilization to maximize crop yield usually results in delayed flowering time (heading stage of rice) and maturity, thereby affecting resource utilization efficiency and subsequent planting time. N-mediated heading date 1 (Nhd1) can directly activate the flowering gene OsHd3a in rice. The inactivation of Nhd1 or OsHd3a leads to delayed flowering time and insensitivity to N supply. The effect of nitrogen application on rice flowering time exhibits genetic diversity, and the single nucleotide polymorphism of the Nhd1 promoter may be related to different responses of flowering time to nitrogen.^96^

Studies have shown that at least 20% of the genes in Arabidopsis are expressed rhythmically, with peaks or troughs during the day or night. This regular oscillation enables plants to adjust to changes in the external environment and respond to endogenous signals.

## The circadian clock participates in responses to drought stress

Plants respond to drought stress via abscisic acid (ABA)-dependent and ABA-independent signaling pathways, with peak expression of genes induced by ABA being concentrated in the daytime.^97^ ABA accumulation shows a circadian rhythm with a peak from the afternoon to the evening.^98,99^ ABA accumulates under drought conditions, and *LHY* overexpression inhibits this accumulation.^98^ Overexpressing *PRR7* increases water loss rate in leaves, while stomatal conductance and leaf water loss are significantly reduced in the *prr9 prr7 prr5* triple mutant.^100,101^ ABA reduces transpiration by promoting stomatal closure. *TOC1*-overexpressing plants have increased stomatal opening and leaf water loss rates, while *TOC1*-RNA interference (RNAi) lines display decreased leaf stomatal opening and leaf water loss rates; sensitivity of stomatal movement in response to ABA is lower in both *TOC1* overexpression and *TOC1*-RNAi plants than in wild-type plants.^102^ During drought stress, the *gi-1* mutant displays lower ABA accumulation than in the wild type, weakened stomatal closure, increased leaf water loss rate, and decreased plant survival. Chromatin immunoprecipitation followed by sequencing (ChIP-seq) revealed that LHY, the morning component of the circadian clock, binds to the promoter regions of ABA biosynthesis and signaling genes to regulate their transcription.^98^ The ABA receptor gene *ABA-BINDING PROTEIN* (*ABAR*) is regulated by the circadian clock, and its expression peaks in the early morning. At night, TOC1 binds to the *ABAR* promoter and modulates its expression.^102^ ABA treatments alter TOC1 levels and affect clock timing. MYB96 directly binds to the *TOC1* promoter in response to ABA.^103^ The basic leucine zipper (bZIP) transcription factor ENHANCED EM LEVEL (EEL) also responds to ABA during drought stress. GI forms a complex with EEL and binds to the promoter of the ABA biosynthetic gene *NINE-CIS-EPOXYCAROTENOID DIOXYGENASE 3* (*NCED3*), promoting its expression (Baek D et al., 2020). Studies in soybean revealed that the absence of all CCA1 and LHY homologs (GmLCLs; CCA1–LHY-LIKE) results in increased stomatal closure under drought conditions, reduced rate of leaf water loss, and enhanced drought tolerance.^7,104^ Drought causes a phase shift in the rhythmic expression of *GmLCL*s. GmLCLs may negatively regulate the response of soybean to drought stress by regulating the circadian rhythmic expression and amplitude of several ABA metabolism and signaling genes. Barley *Ppd-H1* is homologous to Arabidopsis *PRR7*. Spikelet number is reduced and flower development is delayed in spring cultivars containing a *Ppd-H1* variant under mild drought stress.^105^ In the annual herb coyote tobacco (*Nicotiana attenuata*), the function of TOC1 in acclimation to drought stress is organ-specific, and the expression of *TOC1* in stems and leaves plays a critical role in acclimating plants to drought conditions.^106^

## The circadian clock regulates the response to salt stress

The sensitivity of Arabidopsis to salt stress is time-specific, in that NaCl has little effect on stem or leaf growth at night, but shows a significant inhibitory effect in the daytime. In addition, *GI*-overexpressing plants are more sensitive to salt stress, while the *gi-1* mutant, *ELF3*-overexpressing plants, and the *prr9 prr7 prr5* mutant are more tolerant to salt stress.^101,107,108^ The SALT OVERLY SENSITIVE (SOS) signaling pathway is an important mechanism in Arabidopsis for coping with salt stress.^109^ GI, a key component of the circadian clock output pathway, participates in salt stress tolerance by negatively regulating the SOS pathway. Under non-stress conditions, GI interacts with SOS2 to inhibit its function; under salt stress conditions, GI is degraded, and the released SOS2 activates the plasma membrane-localized Na^+^/H^+^ antiporter SOS1 to expel Na^+^ from cells.^107^ ELF3, a key component of the EC, is involved in salt stress tolerance by mediating protein stability and transcription: on the one hand, ELF3 weakens the inhibitory effect of GI on SOS2 by reducing the stability of GI protein; on the other hand, ELF3 promotes the transcription of genes encoding positive regulatory factors and inhibits the transcription of genes encoding negative regulatory factors of salt stress tolerance by inhibiting *PIF4* expression.^108^ Loss of function of the ELF3 homolog GmJ in soybean is associated with decreased salt stress tolerance. The expression of several salt-stress-responsive genes is decreased in the *j* mutant, including genes encoding transcription factors related to salt stress tolerance.^110^ OsPRR73 regulates rice tolerance to salt stress.^111^ In rice, HIGH-AFFINITY K+ TRANSPORTER 2;1 (OsHKT2;1) localizes to the cell membrane and transports Na^+^ into the cell. OsPRR73 binds to the *OsHKT2;1* promoter and inhibits its transcription by recruiting HISTONE DEACETYLASE 10 (HDAC10), thus enhancing salt stress tolerance.^111^

## The circadian clock regulates plant mineral nutrition stress

Homeostasis of essential metal ions is important for maintaining circadian rhythmicity. Circadian period lengthens upon copper deficiency or excess zinc, while the circadian clock is not significantly affected by zinc or manganese deficiency.^112^ The period of the circadian clock is inversely proportional to the concentration of iron or magnesium present in the culture medium.^112,113^ Iron homeostasis in plants is important for avoiding oxidative stress and maintaining growth and development.^114^ The chlorophyll content of the *cca1 lhy* double mutant is sensitive to the iron concentration in the culture medium.^100,115^ In addition, increasing iron content in the medium significantly decreases the chlorophyll content of plants overexpressing *PRR7* when compared with the wild type.^100^ TIC localizes in the nucleus and participates in the maintenance of circadian rhythmicity and amplitude. The T-DNA insertion mutant *tic-2* is sensitive to the iron concentration in the culture medium. The mild chlorosis phenotype of *tic-2* leaves can be restored by applying an iron-containing nutrient solution. TIC is also involved in regulating iron-response genes via the circadian clock.

IRON-REGULATED TRANSPORTER 1 (IRT1) is a high-affinity Fe^2+^ transporter; the *irt1–1* mutant has a long-circadian-period phenotype. Promoter activity of *IRT1* is regulated by the circadian clock and peaks in the early morning.^116^ FERRIC REDUCTION OXIDASE 2 (FRO2) is an iron reductase. CCA1 binds directly to *IRT1*, *FRO2*, and several regulatory regions of multiple iron homeostasis genes, activating their expression. In the *cca1 lhy* double mutant, the activity of the *IRT1* and *FRO2* promoters is decreased and their rhythmic expression is lost.^115^ FERRITIN (FER) plays a crucial role in the response to oxidative stress induced by free iron ions. ChIP-seq results show that *FER1*, *FER3*, and *FER4* are targets of PRR7; their rhythmic expression is inhibited by PRR7, with their lowest expression in the evening and at night.^100^

Magnesium is a cofactor of various enzymes and a critical component of chlorophyll. The concentration of magnesium in human U2OS and *Ostreococcus tauri* cells shows rhythmic oscillations and peaks in the early morning. The balance of energy metabolism in the cells is maintained by Mg^2+^ fluctuations that promote metabolism and translation.^117^ The core oscillator in Cyanobacteria is composed of three proteins: KaiA, KaiB, and KaiC.^118^ Phosphorylation/dephosphorylation of KaiC is dependent on the magnesium ion concentration *in vitro*: low magnesium ion concentrations lead to KaiC phosphorylation, while high magnesium ion concentrations lead to KaiC dephosphorylation.^119^ The effect of magnesium on the circadian cycle in Arabidopsis is dependent on light but does not appear to require active photosynthesis. Magnesium ions may participate in the maintenance of circadian rhythms by affecting protein translation.^113^ Recent studies in rice revealed that the rhythmic expression of *MAGNESIUM TRANSPORTER 3* (*OsMGT3*), which encodes a magnesium transporter located in the chloroplast envelope, peaks in the early morning.^120^

## Conclusions and future perspectives

Abiotic stress signals are often temporary or temporally gated across the day; for example, high temperature and water stress usually occur during the daytime, while low temperature stress will most likely occur at night. The circadian clock receives environmental signals and makes adjustments to maximize plant growth and development in response to changing external environments. We discussed in this review how the core components of the circadian clock participate in various plant responses to environmental signals such as light, temperature, drought, salt, and mineral nutrition, and highlighted how mutations in core circadian clock genes can alter the plant response to abiotic stresses. For example, CCA1 and LHY regulate tolerance to stresses such as iron deficiency and freezing, while RVE4 and RVE8 regulate tolerance to freezing and high-temperature stresses.^43,121–123^ PRRs regulate tolerance to low temperature, drought, and salt stresses.^55;100–102^ The EC plays an important role in regulating the response to high temperature and salt stresses;^108,124^ and GI, a key component of the circadian clock output pathway, is involved in regulating the response to freezing, drought, and salt stresses (Figure 1);^107;108;125^ Baek, 2020).

The circadian clock confers tissue and organ specificity in its regulation of plant stress tolerance. The function of TOC1 in *N. attenuata* in the response to drought was investigated separately in shoots and roots.^106^ ELF4 is transported from shoots to roots in response to temperature changes.^126^ Using tissue- and organ-specific promoters will aid our understanding of circadian clock-mediated regulation of the stress response at the tissue and organ level, which could improve stress tolerance in crops. Clustered regularly interspaced short palindromic repeats (CRISPR)/CRISPR-associated protein 9 (Cas9)-mediated gene editing can be used to mutate circadian clock-related *cis*-acting elements in the promoter regions of stress tolerance genes to confer stress tolerance.

Research on the circadian clock and circadian rhythms will facilitate cultivar selection and improve crop productivity and quality. Key circadian clock genes play important roles in crop growth and development and environmental acclimation. For example, increased nighttime temperatures during the reproductive growth stage of rice alter the circadian expression patterns of many genes, leading to decreased yield and quality.^127^ CCA1 and LHY homologs in soybean negatively regulate drought tolerance,^7,104^ while the ELF3 homolog J positively regulates salt stress tolerance.^110^ OsPRR73 positively regulates salt stress tolerance in rice,^111^ the PRR7 homolog Ppd-H1 in barley contributes to drought tolerance,^105^ and GI negatively regulates freezing tolerance in *Brassica rapa*.^128^ Many regulatory components and mechanisms have been discovered and analyzed through the in-depth study of circadian rhythms and abiotic stress responses, laying a theoretical foundation for optimizing the balance between crop growth and development and the stress response and for breeding superior germplasm.
